# The effect of religious belief on Chinese elderly health

**DOI:** 10.1186/s12889-020-08774-7

**Published:** 2020-05-06

**Authors:** Yucheng Chen, Yuxiao Zhao, Zengwen Wang

**Affiliations:** The Research Center of Social Security, Wuhan University, Wuhan, China

**Keywords:** Elderly health, Religious belief, DID-PSM, China

## Abstract

**Background:**

With the accelerated ageing of the population in China, the health problems of elderly people have attracted much attention. Although religious belief has been shown to be a key way to improve the health of elderly people in various studies, little is known about the causal relationship between these variables in China. This paper explores the effect of religious belief on the health of elderly people in China, which will provide an important reference for China to achieve healthy ageing.

**Methods:**

Balanced panel data collected between 2012 and 2016 from the China Family Panel Studies (CFPS) were used. Health was assessed using self-rated health, and religious belief was measured by whether the respondents believed in a religion. The DID+PSM method was employed to solve the endogeneity problem caused by self-selection and omitted variables. In addition, the CESD score (replacing self-rated health) and different matching methods (the method of PSM after DID method) were used to perform the robustness test.

**Results:**

The results show that religious belief has no significant effect on the health of elderly people. With the application of different matching methods (one-to-one matching, K-nearest neighbour matching, radius matching and kernel matching) and replacing the health indicator (the CESD score) with the above matching methods, the results are still robust.

**Conclusion:**

In China, religious belief plays a limited role in promoting “healthy ageing”, and it is difficult to improve the health of elderly people only via religious belief. Therefore, except for focusing on the guidance of religion with regard to healthy lifestyles, multiple measures need to be taken to improve the health of elderly people.

## Background

The health of elderly people has become a worldwide issue in the context of ageing. At present, the ageing of China’s population has become more serious; specifically, the number of people aged 65 and above in China increased from 90 million in 2001 to 158 million in 2017.^1^ With increasing age, the physical function of elderly people gradually declines, and their morbidity is much higher than that of younger people. As a result, ageing will have a greater impact on the costs of health care and medical insurance, which will bring a heavy financial burden to society as a whole. Against this background, guaranteeing and maintaining the health of elderly people is essential for China to cope with ageing. At the same time, there exists evidence showing that religious belief is related to health, and it is worth analysing the health of elderly people from the perspective of religious belief.

In recent years, religious fervour has increased in China, and the proportion of people with religious beliefs has risen rapidly. By 2018, the number of people with religious beliefs in China reached approximately 200 million, accounting for nearly 12.8% of the total population.^2^ Religion originates from the fear of death, and death is closely related to people’s health. Therefore, the relationship between religious belief and health needs to be further explored, as understanding this relationship may lead to an effective way to promote healthy ageing.

A large number of studies have focused on the relationship between religious belief and health [8, 16]. Theoretically, the relationship between these variables is ambiguous. On the one hand, religious belief can improve health, as some religious dogmas promote healthy lifestyles and behaviours; for example, Mormonism strictly prohibits smoking and drinking [14]. On the other hand, some religious dogmas may be harmful to health, as they are in conflict with medicine. For example, patients with religious beliefs may think that their lifespan is determined by a god and thus refuse medical treatment. Moreover, many pathological declines are unacceptable to religious believers [32, 33]. Blanchard et al. [2] found that Catholics and mainstream Protestants have lower death rates because their faiths encourage investment in public goods. The situation is different in Conservative Protestants, as the otherworldly orientation of their faith decreased people’s willingness to invest in public health. Thus, different religions and dogmas will have different effects on health.

The results of empirical studies on the relationship between religious belief and health are also mixed. Some studies have found that religious belief has a significant positive effect on health [4, 5, 10, 14, 21, 30]. In contrast, others have found that the effect of religious belief on health is significantly negative [2, 3, 12, 33]. Thus, there are multiple complex mechanisms underlying the influence of religious belief on health. Existing studies have suggested several factors. The first is behaviour, in which religious belief influences health by changing people’s health-related behaviours [12, 15, 18, 26, 28]. Second, social support and religious belief can improve the health of believers by increasing social participation and broadening social networks [4, 15, 17, 25, 30]. The third factor is healthcare utilization because some religious dogmas can influence believers’ attitudes towards healthcare utilization [1, 2, 12]. The fourth is the psychological factor. Religious belief can regulate emotion and relieve stress by cultivating people’s faith and advocating forgiveness [4, 14, 17, 30, 34]. The final factor is mysterious power, which means that the effect of religion on health is a mysterious power rather than a secular mediating factor [7]; however, this factor is difficult to measure and analyse.

Previous studies have examined the potential underlying mechanism between religious belief and health, and they have also provided important references for analysing the effect of religious belief on the health of elderly people in China. However, these studies also have certain shortcomings. First, most studies focus on Western countries, while studies on East Asian countries (such as China) are rare. Second, the data used in most of these papers are cross-sectional data, which makes it difficult to identify the causal relationship between religious belief and health [37]. One challenge in identifying the causal relationship between these variables is that religious belief is endogenous to health. In other words, health status will affect people’s decision regarding whether to be a believer and whether to participate in religious activities [1]. Third, unobservable missing variables may affect religious belief and health at the same time, which may lead to the bias of estimation results.

China is greatly influenced by traditional Buddhism, Taoism, and Confucianism, and the impact of religion on health in China may also be different from that in Western countries. Therefore, the conclusions about Western countries may not apply to China due to cultural differences. Moreover, most of the previous literature failed to recognize the causality between these variables because of endogeneity problems. Although Jiang et al. [14] tried to explore the effect of religious belief on elderly health in China by using panel data, the data they used are from 2002 to 2005 [15]. Therefore, these data are too old to reflect the current reality of China. Additionally, the proportion of believers in China is much lower than that in Western countries, which may also influence the estimation results. Therefore, against the background of ageing in China, exploring the causal relationship between religious belief and the health of elderly people not only fills a gap in the existing research but also provides practical information for improving the role of religion in healthy ageing.

The objective of this paper is to estimate the effect of religious belief on the health of elderly people in China by using panel data collected between 2012 and 2016 from the China Family Panel Studies (CFPS). To address endogeneity problems, the combination of difference-in-difference (DID) and propensity score matching (PSM) is used to eliminate the effect of time-invariant and unobservable factors and self-selection^3^ on the estimation results. To solve the reverse causality between religious belief and the health of elderly people, we also control for the respondents’ baseline health status (the health status of respondents in 2012) in the model. According to the experiences of existing studies and the potential influence mechanism of religious belief on health, we propose the following research hypothesis:

Religious belief has a significant positive effect on the health of elderly people, as the dogmas of five major religions (Buddhism, Taoism, Islam, Protestantism, Catholicism) in China may promote healthy lifestyles and provide comfort and a social support network for believers.

This paper proceeds as follows. Section 2 describes the data and empirical model. Section 3 presents the empirical results. Section 4 includes the discussion, conclusions and policy suggestions.

## Method

### Data

This paper uses data collected between 2012 and 2016 from the China Family Panel Studies (CFPS). The CFPS is a nationally representative longitudinal tracking survey conducted by the Institute of Social Science Survey (ISSS) of Peking University. The subsample frame of the CFPS was obtained via a three-stage (districts/counties - villages/communities - households) probability of random sampling, and the samples covered 621 villages/communities from 25 of China’s 30 provinces. The data were collected through computer-assisted personal interviews (CAPI). The baseline survey of the CFPS started in 2010, and the fourth follow-up was completed in 2016. The questionnaire assesses detailed information on the health, religious belief, socioeconomic characteristics and other factors of communities, households and individuals.

Although the CFPS started in 2010, certain indicators used in 2010 changed in 2012 and in later surveys. Therefore, we take the data in 2012 as the baseline to keep the indicators consistent. As the data used in this paper are balanced panel data collected between 2012 and 2016; the samples that were lost or newly added during this period were deleted. In this paper, people with no religious belief in 2012 but with religious belief in 2016 are treated as the treatment group. People without religious belief throughout the study period are treated as the control group. Additionally, respondents under 60 were also excluded because the aim of this paper is to study the health of elderly people. In this process, the sample size changed as follows: the number of respondents in the balanced panel data between 2012 and 2016 was 27,209, and after excluding the respondents under 60 years old, 10,918 were left. Then, after excluding the respondents with missing variables, the final analytical sample was 8044.

### The definition of the variables

#### Dependent variable

The dependent variable is the health status of the respondents, and it is mainly measured by self-rated health. Self-rated health has proven to be an effective and reliable indicator that measures individuals’ cognitive ability, morbidity and mortality and comprehensively reflects their physical and mental health status [19, 27]. The CFPS questionnaire asked the respondents to rate their health status. The score ranges from 1 to 5, and the higher the score is, the worse the self-rated health status. The difference in self-rated health between 2012 and 2016 shows the change in health, and it was used for the first step of the difference-in-difference (DID) technique. When the change in health is negative, it means that health has improved. In contrast, a positive change indicates that health has deteriorated.

However, there exists a problem in that the same value of health change may have different meanings. For example, when the value is − 1, it may mean that the health of an individual changed from very healthy to relatively healthy or from healthy to fair. Although the value is the same, the change in health is different. Against this backdrop, in accordance with the practice of Sun and Wang [30], we redefined the change in health to perform a robustness test [31]. Specifically, when health is improved, the change is recoded as − 1; when health is deteriorated, the change is recoded as 1; and 0 means no change in health.

In addition, depression symptoms (the mental health of elderly people) were also used to perform the robustness test. Depression symptoms were measured by the Center for Epidemiologic Studies Depression (CESD) scale. The CESD scale has proven to be reliable and effective in measuring mental health, as the design of the scale has internal consistency and sufficient repeatability [22]. The total score of the CESD scale ranges from 0 to 60. The higher the score is, the more severe the depressive symptoms. In this paper, a score of 21 points was used as the cutoff for depressive symptoms [28].^4^ Individuals are considered to have depression symptoms if their CESD score is 21 and above. The difference in depression symptoms (CESD_d) was defined as follows: if the respondents scored 21 or above in 2012 and lower than 21 in 2016, it was considered that the depression symptoms had been improved (recoded as − 1); if the respondents scored lower than 21 in 2012 and 21 or above in 2016, it was considered that the depression symptoms had been worsened (recoded as 1); if the score was lower than 21 or 21 or above in both 2012 and 2016, depression symptoms were considered unchanged (recoded as 0).

#### Independent variable

Religious belief is the independent variable in this paper. However, there is currently no consensus on the measurement of religious belief in academia. For example, in the previous literature, religious belief was measured by an individual’s attitude towards religion, whether an individual belongs to a religion or the frequency of participation in religious activities [14, 26, 34]. Some studies also measure religious belief by assessing how much individuals volunteer in churches, read religious books and watch religious television programmes [6, 20]. Considering the lower proportion of believers in China and the availability of data, we measure religious belief based on whether the respondent believes in a religion. The CFPS used in 2012 asked the respondents what religion they belonged to. The answers included seven options: “Buddhism”, “Taoism”, “Islam”, “Protestantism”, “Catholicism”, “no religion” and “other”. We treat the individuals who belong to one of these religions as believers (coded as 1) and those who answered “no religion” or “other” as non-believers (coded as 0).

#### Control variables

The health of elderly people is also influenced by a series of individual demographics, household characteristics and socioeconomic factors [11, 14]. In this paper, we controlled for the following variables: age, sex, marital status, ethnicity, baseline health, family size, household registration, educational level, average household income, medical insurance, drinking water, time to the nearest clinic and region. Additionally, evidence suggests that religious belief is also affected by individual demographic characteristics, health status and socioeconomic factors [13, 35, 36]. Therefore, the above variables are also covariates that influence decision-making related to religious belief. The specific definitions of the variables are shown in Table 1.

**Table 1:** The description of variables

Variables	Description
Dependent variables	
SRH	Self-rated health, very healthy = 1/ relatively healthy = 2/ healthy = 3/ fair = 4/ unhealthy = 5
CESD	It includes 20 kinds of feelings and behaviors, every kind of feelings and behaviors have been divided into 0 (barely) to 3 (always) point
Independent variables	
Religious belief	Buddhism/Taoism/Protestant/Catholicism/Islam = 1; Otherwise = 0
Control variables	
Age	The age of the respondent
Gender	Female = 0; Male = 1
Marital status	Married or cohabitation = 1; Single, divorced or widowed = 0
Ethnicity	Han ethnicity = 1; Ethnic minorities = 0
Baseline health	The self-rated health of respondents in 2012
Family size	The number of family members
Household registration	Urban = 1; Rural = 0
Educational level	
Illiterate	Illiterate = 1, others = 0
Primary school	Primary school = 1, others = 0
Junior middle school	Junior middle school = 1, others = 0
Senior middle school	Senior middle school = 1, others = 0
College and above	College and above = 1, others = 0
The average household income	The log of per capita net income of the household
Medical insurance	One of free medical insurance, urban employee basic medical insurance, urban resident basic medical insurance, supplementary medical insurance or new cooperative medical scheme = 1; Otherwise = 0
Water	The source of drinking water is from tap water/mineral water/purified water/filtered water = 1; otherwise = 0
Time	The time of the fastest way to the nearest medical point (Unit, minute)
Region	
Eastern	Eastern = 1, others = 0
Central	Central = 1, others = 0
Western	Western = 1, others = 0

### Descriptive statistics and model

We first performed a descriptive statistical analysis of the variables in this paper. For dependent and independent variables, we reported the proportion of religious believers and non-believers and their median self-rated health scores, respectively. With respect to control variables, we reported the mean for continuous variables and the percentages for categorical variables. The maximum and minimum values of all control variables are also presented.

The model used in this paper is a combination of the difference-in-difference (DID) technique and propensity score matching (PSM). Because believing in religion is a self-selection behaviour rather than a random distribution, this may lead to biased estimates. In addition, there also exist certain unobservable factors that will affect the results. Against this backdrop, it is difficult to identify the causal relationship between the health of elderly people and religious belief. PSM can solve the problem of self-selection by matching the control to the treatment based on a series of observable variables. However, the average treatment effect on the treated (ATT) is also biased because it cannot identify the effect of unobservable factors on health. DID can overcome the limitation of PSM by eliminating the influence of time-invariant and time-variant synchronistic unobservable factors on the results. PSM can also ensure that DID meets the common trend assumption to a certain extent. Therefore, DID+PSM can solve the endogeneity problems caused by self-selection and unobservable factors.

Specifically, 1) we subtract the health score in 2012 from the health score in 2016 in the treatment and control groups; then, 2) we use the difference between the health of the two periods as the dependent variable and estimate the average treatment effect on the treated individuals based on a series of matched variables in the base period. The model is as follows:

$$ {ATT}_{DID- PSM}=E\left[{Y}_{1i}^T-{Y}_{0i}^T|P\left({X}_{0i}\right),{D}_i=1\right]-E\left[{Y}_{1i}^C-{Y}_{0i}^C|P\left({X}_{0i}\right),{D}_i=0\right] $$

where D represents the dummy variable of religious belief (believer = 1, non-believer = 0). T indicates the treatment group. C indicates the control group. Y_1i_ is the health status of individual i in 2016, and *Y*_0*i*_ is the health status of individual i in 2012. P is the probability of individuals entering the treatment group or the control group. One-to-one matching, K-nearest neighbour matching, radius matching and kernel matching were used to match the treatment and control groups. In this model, health is the amount of health change between 2012 and 2016 rather than the health status at a certain point in time [9]. The details about DID and PSM can be found in the appendix.

Additionally, individuals’ health status may affect their decision-making on religious belief. To solve the endogeneity problem caused by reverse causality, we also controlled for the individuals’ health status at baseline.

## Results

### Statistical analysis

Table 2 presents the distribution of self-rated health in the treatment and control groups. From Table 2, we can see that the believers account for 8.70% of the samples. From 2012 to 2016, the median self-rated health of the treatment and control groups was 4, which indicates that there is no obvious difference in the health of elderly people between the two groups. However, this is only a statistical description, and further empirical tests are needed.

**Table 2:** The descriptive statistics of dependent and independent variables

	2012	The median of SRH	2016	The median of SRH
Treated	350	4	350	4
	(8.70%)		(8.70%)	
Controlled	3672	4	3672	4
	(91.30%)		(91.30%)	
Total	4022		4022	

Notes: SRH is the self-report health. The values in brackets are the percentage of treated or the controlled

Table 3 reports the statistical results of the control variables. The average age of the respondents is approximately 66, indicating that most of the respondents are younger elderly people. Consistent with the characteristics of younger elderly people, 83.4% of them have spouses or cohabitate. More than half of the sample are males, and 94.6% are of Han ethnicity. The mean baseline health score is 3.708, and the average family size is 3.851. Approximately 44.5% of the respondents live in urban areas. Their educational level is generally low, and the proportion of illiterate individuals is approximately 53.3%. The average logarithm value of household per capita income is 8.738. Approximately 90% of the respondents are covered by medical insurance, and the average minutes to the nearest medial institution is 12.875. A total of 64.5% of the respondents drink tap water. With respect to region, nearly half of the respondents live in eastern China (45.5%), followed by central China (28.9%), with the fewest respondents living in western China (25.6%).

**Table 3:** Control variables and its influence on religious belief

Variables	Mean/Percentage	Min	Max	Coef.	95% CI
Age	66.772	60	90	−0.001	(− 0.021, 0.020)
Gender	53.3%	0	1	−0.475^***^	(−0.711,-0.239)
Marital Status	83.4%	0	1	−0.108	(−0.409,0.194)
Ethnicity	94.6%	0	1	0.314	(−0.327,0.955)
Baseline Health	3.708	1	5	0.029	(−0.074,0.132)
Family Size	3.851	1	14	0.035	(−0.019,0.089)
Household Registration	44.5%	0	1	0.302^**^	(0.059,0.544)
Educational level					
Illiterate (ref)	53.3%	0	1	/	/
Primary school	24.4%	0	1	0.051	(−0.224,0.327)
Junior middle school	13.9%	0	1	−0.317	(− 0.698,0.064)
Senior middle school	5.8%	0	1	−0.280	(−0.833,0.272)
College and above	2.6%	0	1	−0.596	(−1.535,0.343)
The average household income	8.738	1.466	12.850	−0.113^**^	(−0.203,-0.024)
Medical Insurance	89.8%	0	1	0.267	(− 0.121,0.656)
Water	61.5%	0	1	0.054	(−0.201,-0.308)
Time	12.875	1	300	0.004	(−0.002,0.011)
Region					
East (ref)	45.5%	0	1	/	/
Central	28.9%	0	1	−0.156	(−0.415,0.104)
West	25.6%	0	1	−0.667^***^	(−0.989,-0.346)
_cons	/	/	/	−1.701^*^	(−3.567,0.165)
Obs	4022				
Pseudo R^2^	0.026				

Notes: * *p* < 0.1; ** *p* < 0.05; *** *p* < 0.01. The covariates used in this part are all from the base period. In the second column, mean corresponds to continuous variables and percentage corresponds to dummy variables. SD CI is the confidence interval

### Propensity score estimation

The first step of PSM is to estimate the propensity score. The propensity score denotes the conditional probability of respondents entering the treatment group given multidimensional feature variables [23]. In this paper, the decision on religious belief is considered to be affected not only by individual characteristics but also by family-related and socioeconomic factors. Thus, we construct a logit model to estimate the propensity score of an individual’s religious belief with as many covariates as possible. The equation is as follows.

$$ logit\left( treated=1\right)={\beta}_0+{\beta}_1X+\varepsilon $$

In this equation, “treated” is a dummy variable that represents whether an individual had religious belief in 2016 but not in 2012. Based on this equation, the probability of religious belief is predicted. *X* represents covariates that affect an individual’s religious belief and health, including age, sex and educational level. The estimation results are presented in Table 3.

### Balance test of matching quality

After estimating the propensity score, we performed a balanced test for the quality of propensity score matching. In other words, we tested whether the covariates had significant differences between the treatment and control groups. Theoretically, under the conditional exogenous hypothesis, all covariates are balanced between the two groups and there are no systematic differences in their distribution. In this paper, the matching quality is indicated by the standardized bias. The results show that there are no significant differences in any covariates between the treatment and control groups after matching. In addition, the *p* value of the combined test also shows that the propensity score of the combined distribution is the same between the two groups, which indicates that the quality of PSM is good (the details of the matching results can be seen in Table 1).

### Average treatment effect of one-to-one matching

The one-to-one matching method was used to analyse the effect of religious belief on the health of elderly people. As Table 4 shows, there is no significant difference in health changes between the treatment and control groups before matching (similar to not controlling for other variables but only using DID). However, the results after matching also show that religious belief has no significant effect on the health of elderly people (the result of DID+PSM), which is inconsistent with the hypothesis of this paper.

**Table 4:** The effect of religious belief on the health of the elderly (One-to-one matching)

	SRH_d1			S.E.	Common support	
	Treated	Controls	ATT		Treated	Controls
Unmatched	−0.109	− 0.093	−0.015	0.068	350	3672
Matched	−0.109	−0.077	− 0.031	0.110	350	349

Notes: SRH_d1 is the difference of self-rated health score between 2012 and 2016. ATT is the average treatment effect on the treated. S.E. is standard error. The S.E. of matched ATT is estimated by bootstrap (with 500 replications)

### Robust test

#### Redefining the change in self-rated health and using different matching methods

As there exists a problem in which the same value may represent different meanings for SRH_d1, we test the robustness of the results by redefining the change in self-rated health (SRH_d2) first. The details of SRH_d2 are described in the Methods section. Additionally, based on the variable SRH_d2, we then use different matching methods (one-to-one matching, K-nearest neighbour matching, radius matching and kernel matching) to perform the robustness test. Table 5 presents the average treatment effect on the treatment group after redefining the change in self-rated health and using different matching methods. The results are similar to those in Table 4. After one-to-one matching, the health of elderly people in the treatment and control groups was not significantly different. The same results were observed for K-nearest neighbour matching, radius matching and kernel matching methods. These results also indicate that the effect of religious belief on the health of elderly people is not significant.

**Table 5:** The effect of religious belief on the health of elderly people (SRH_d2)

Matching method	Matching parameter	Common support sample size	SRH_d2			S.E.
			Treated	Controls	ATT	
One-to-one matching	k = 1	699	−0.051	−0.066	0.014	0.067
K-nearest neighbour matching	k = 10	4016	−0.051	−0.044	− 0.007	0.047
Radius matching	δ=0.01	4015	−0.052	−0.064	0.012	0.037
Kernel matching	k: epan bw: 0.06	4016	−0.051	−0.061	0.009	0.038

Notes: ATT is the average treatment effect on the treatment group. S.E. is the standard error estimated by bootstrapping (with 500 replications). K is the number of neighbourhoods for matching; δ is the matching radius; Epan is the kernel function; Bw is the bandwidth

#### Replacing health indicators and using different matching methods

In this section, we test the robustness of the results by replacing the health indicator and using different matching methods. The existing studies found that religious belief can provide comfort and lift anxiety and fear, and it may have a significant effect on the mental health of elderly people [4, 6, 14]. Therefore, self-rated health was replaced by the Center for Epidemiologic Studies Depression (CESD) in this section, and the different matching methods (one-to-one matching, K-nearest neighbour matching, radius matching and kernel matching) were used to perform the robustness test. As Table 6 shows, the ATT of one-to-one matching and K-nearest neighbour matching are not significant. Although the ATT values of radius matching and kernel matching are significant, they are only significant at the 10% level and in opposite directions. As a result, there is insufficient evidence to show that religious belief has a significant effect on the mental health of elderly people in China, and the hypothesis of this paper has not been verified.

**Table 6:** The effect of religious belief on the health of the elderly (CESD_d)

Matching method	Matching parameter	Common support sample size	CESD_d			S.E.
			Treated	Controls	ATT	
One-to-one matching	k = 1	699	−0.054	−0.066	0.011	0.040
K-nearest neighbor matching	k = 10	4016	−0.054	−0.059	0.004	0.032
Radius matching	δ=0.01	4015	−0.054	−0.057	0.002^*^	0.030
Kernel matching	k: epan bw: 0.06	4016	−0.054	−0.052	− 0.003^*^	0.029

Notes: *p < 0.1. CESD_d is the improvement of self-report depression scale between 2012 and 2016. ATT is the average treatment effect on the treated. S.E. is standard error that estimated by bootstrap (with 500 replications). K is the number of neighborhoods for matching; δ is the matching radius; Epan is the kernel function; Bw is the bandwidth

## Discussion

This paper estimates the effect of religious belief on the health of elderly people in China. We find that there is no evidence that religious belief significantly improves the health of elderly people. The result is still robust after replacing the health indicators (from self-rated health to CESD) and using different matching methods. The reason may be that most elderly believers believe in Chinese traditional Buddhism and Taoism. Although Buddhism and Taoism teach followers to achieve healthy longevity and immortality through self-cultivation, believers are more likely to pray for good health and luck rather than change their unhealthy lifestyles. In addition, there is less communication and fewer collective activities among believers in traditional Chinese Buddhism and Taoism, so it is also difficult to form a social support network based on religion. The results of this paper also indicate that the role of religion in achieving healthy ageing is limited.

Our findings contrast with the existing studies using data from other countries. Those studies tend to find significant positive or negative correlations between religious belief and health [5, 20, 24, 29]. The reasons can be explained as follows: First, the previous studies in other countries focus on the correlation between religion and health instead of the causal relationship, which may lead to different results. Second, the difference in religious dogmas may lead to different results. In Western countries, most believers belong to Catholicism, Protestantism and Islam, and the binding forces are relatively stronger. In China, traditional Buddhism and Taoism believers account for a large proportion, and the binding forces are relatively weaker. For example, the Catholic dogma holds that life and death, as well as whether one is rich or poor, are all determined by God. This fatalism may affect believers’ enthusiasm for disease treatment. Meanwhile, in Protestantism, the concept of original sin may make believers feel guilty and thus affect their health. However, the ban on premarital sex promoted by both religions also helps reduce the incidence of AIDS. Third, there exist differences in social capital among believers in different countries. In Western countries, religious churches can help believers to know each other and to establish social support networks by organizing religious activities. In this case, believers can acquire material, information and medical support to maintain their health. In China, except for professional religious staff (such as monks), people are individual believers, they have fewer connections with each other, and it is difficult for them to form a large social support network.

The results are also associated with the development stage of China. Since the reform and opening up, China has made great progress in economic development, but imbalances and inadequacies still exist in different groups and regions. More people in China pursue material wealth than spiritual wealth. Moreover, people have the freedom to believe in religion in China. Against this background, religious belief is more based on instrumental rationality than value rationality in China [34]. Additionally, with the deepening of China’s reform and opening up, the uncertainty caused by the market economy has increased people’s various risks, and formal risk-sharing mechanisms are lacking due to the relatively lagging development of the social security system. In this case, religious belief has become an informal way to spread risk [13, 35]. The main aim of elderly Chinese people with religious belief is to reduce financial risk, which is particularly common in rural China [34]. Therefore, the capacity of religious belief in China to improve the physical and mental health of elderly people is extremely limited.

This paper also has some limitations. It is difficult to distinguish the health effects of different religions because of the availability of data. In addition to the considerable differences between Chinese traditional religions and other countries’ religions, there are also differences in the historical background and dogmas among different religions in the same country. As a result, their health effects are also different. However, in this paper, the number of religious believers is only 350. Among them, those who believe in Buddhism accounted for approximately 77.86%; Protestantism accounted for approximately 12.86%; and Catholicism and Islam only accounted for 4 and 0.57%, respectively. Therefore, this paper could not analyse the specific health effects of different religious beliefs, which is an important direction for future studies.

## Conclusion

With the acceleration of ageing in China, the health problems of elderly people have attracted considerable attention. Elderly people face health problems caused by increasing diseases, declining physical function and reducing social networks and support. They need to maintain their health via a series of measures, such as leisure, exercise, and medical activities. Religious belief is also an important way for elderly people to increase their health. This paper uses data collected between 2012 and 2016 from the CFPS to estimate the effect of religious belief on the health of elderly people in China. The DID+PSM method is used to solve the endogeneity problem caused by self-selection and omitted variables. The results show that religious belief has no significant effect on the health of elderly people, and this finding is still robust after a series of tests. This result indicates that religious belief plays a limited role in promoting “healthy ageing”, and it is difficult to improve the health of elderly people only through religious belief. Therefore, multiple measures need to be taken to improve the health of elderly people. As the Chinese government implements policy on the freedom of religious belief, believers can adhere to relevant dogmas to form a healthy lifestyle. For non-believers, on the one hand, individuals should build health awareness and take responsibility for their own health. On the other hand, the government should take measures to comprehensively intervene in health-related factors, improve the medical security system and guide elderly people to prevent and treat diseases.

## Data Availability

The datasets we used in this paper were from the China Family Panel Studies (CFPS). This study was carried out by the Institute of Social Science Survey, Peking University (ISSS), which is a national, large-scale and multidisciplinary follow-up survey project. This project was funded by Peking University and the Chinese National Natural Foundation. All participants in the survey provided written informed consent. The data were released to the researchers without access to any personal data. The link online is http://www.isss.pku.edu.cn/cfps/index.htm. The datasets generated and/or analysed during the current study are not publicly available for reasons of confidentiality but are available from the corresponding author on reasonable request.

## Abbreviations

CFPS: China Family Panel Studies
DID: difference-in-difference
PSM: propensity score matching
ATT: average treatment effect on the treated
CESD: Center for Epidemiologic Studies Depression
AIDS: Acquired Immune Deficiency Syndrome

## Footnotes

1 The data are from the National Bureau of Statistics of China.

2 The data are from the “White Book on China’s Policy and Practice of Guaranteeing Freedom of Religion”.

3 This ensures that the treatment and control groups are consistent in factors other than religious belief.

4 Shean and Baldwin [28] have proven that self-rated depression at a cutoff score of 21 can identify depressed individuals with sensitivity and specificity rates of 73 and 96.1%, respectively.
